# A nationwide participatory programme to measure person‐centred hospital care in Italy: Results and implications for continuous improvement

**DOI:** 10.1111/hex.13231

**Published:** 2021-05-20

**Authors:** Flavia Cardinali, Sara Carzaniga, Giorgia Duranti, Barbara Labella, Alessandro Lamanna, Micaela Cerilli, Giovanni Caracci, Fabrizio Carinci

**Affiliations:** ^1^ Italian National Agency for Regional Health Services (AGENAS) Roma Italy; ^2^ University of Bologna Bologna Italy

**Keywords:** citizen engagement, monitoring, participatory evaluation, performance assessment, person‐centred care, quality improvement

## Abstract

**Background:**

Patient‐centredness has been targeted by the Italian government as a key theme for the future development of health services.

**Objective:**

Measuring patient‐centred health services in partnership with citizens, health professionals and decision makers.

**Design:**

National participatory survey in a large test set of hospitals at national level.

**Setting and participants:**

A total of 387 hospital visits conducted in 16 Italian regions by over 1,500 citizens and health professionals during 2017‐2018.

**Main variables and outcome measures:**

An ad hoc checklist was used to assess person‐centredness in hospital care through 243 items, grouped in 4 main areas, 12 sub‐areas and 29 person‐centred criteria (scored 0‐10). GEE linear multivariate regression was used to explore the relation between hospital characteristics and person‐centredness.

**Results:**

Person‐centred scores were moderately high, with substantial variation overall (median score: 7.0, range: 3.2‐9.5) and by area (Care Processes: 6.8, 2.0‐9.8; Access: 7.4, 2.7‐9.7; Transparency: 6.7, 3.4‐9.5 and Relationship: 7.3, 0.8‐10.0). Multivariate regression found higher scores for increasing volumes of activity (quartile increase: +0.21; 95% CI: 0.13, 0.29) and lower scores in the south and islands (−1.03; −1.62,‐0.45).

**Discussion:**

The checklist has been applied successfully by over 1,500 collaborators who assessed hospitals in 16 distinct Regions and Autonomous Provinces of Italy. Despite an overall positive mark, all scores were highly variable by location and hospital characteristics.

**Conclusion and patient or public contribution:**

A national participatory programme to improve patient‐centredness in Italian hospitals highlighted critical areas with the direct input of citizens.

## 1 INTRODUCTION

The goal of person‐centredness has become increasingly popular in the organization of health services.

Since its initial definition as one of the six main pillars of quality of care,^1^ various concepts and models have been introduced to assess it routinely,^2^, ^3^, ^4^, ^5^, ^6^, ^7^, ^8^, ^9^ using specific tools to monitor the personal domains of physical, psychological and social needs in primary, secondary and tertiary clinical settings.^10^, ^11^

Relevant experiences addressed the importance of citizen involvement in the direct evaluation of services, embracing the concept of end‐user co‐design, particularly in hospitals.^12^, ^13^, ^14^, ^15^, ^16^, ^17^, ^18^

Following these experimental initiatives, international organizations have recently recognized the role of person‐centredness as a key driver for the sustainability of health systems.

In 2016, the WHO called on Member States to promote an approach to care that ‘consciously adopts individuals', carers', families' and communities' perspectives as participants in, and beneficiaries of, trusted health systems that are organized around the comprehensive needs of people’.^19^, ^20^

At the same time, the OECD released a new framework for performance evaluation including patient‐centredness as one of the tree key dimensions of quality,^21^ presented as the main theme in the Ministerial Conference where Member States agreed that ‘health‐care systems need to engage patients as active players in improving health care’.^22^

Relevant developments took place in parallel to strengthen the Italian National Health System (Sistema Sanitario Nazionale, SSN).

In 2012, the Italian Ministry of Health and the Regions and Autonomous Provinces agreed common terms for the accreditation of health‐care facilities, incorporating patient‐centred care as an essential quality criterion.^23^ Two years later, building upon the OECD recommendation of increasing the direct participation of citizens in quality assurance,^24^ the Ministry of Health signed the ‘Pact for Health 2014‐2016’ with all Regions and Autonomous Provinces. The agreement included the implementation of specific interventions to foster patient‐centred care across the country in a balanced way, calling upon the National Agency for Regional Health Services (AGENAS) to develop a set of core indicators to monitor the results of these interventions. At the same time, relevant activities of patient involvement were carried out by different Regions and Autonomous Provinces. These developments increased the need of improving the comparability of person‐centred care at national level.^25^

Consequently, AGENAS defined a set of materials and protocols to undertake a national survey on person‐centredness in Italian hospitals.

In this paper, we present the results of this activity, focussing on the following research questions:

- Can we measure person‐centredness at hospital level using the same standardised tool across the country, with the active participation of citizens, health professionals and decision makers?
- Which features of person‐centredness are widely applied, and which others deserve increased attention? Is there any significant variation across the country and/or potential association with hospital characteristics?

## 2 MATERIALS AND METHODS

The study was carried out between 2016 and 2018 in the context of a multi‐year programme financed with infrastructural funds available from the general mandate assigned by the Ministry of Health to AGENAS.

### 2.1 Governance of the programme

The project stems from a collaboration started in 2011, when AGENAS established a Project Team, an Advisory Board and the Regional Network of experts to agree on a common definition of patient centredness.

The Project Team formed at AGENAS included authors of this paper with a multidisciplinary background in medicine, public health, psychology, sociology and biostatistics. The role of the Project Team was to coordinate the conduct of a national survey to measure person‐centredness in Italian hospitals through the use of a standardized assessment tool. For executing the project, the Project Team cooperated with the Regional Network, including 34 representatives of Regions and Autonomous Provinces involved in activities of patient empowerment, and the Advisory Board, including experts in the field of civic evaluation of health services from the non‐profit consumer organization ‘Cittadinanzattiva’. The lists of members of the Advisory Board and Regional Network are included in the acknowledgements section together with their affiliations.

In August 2011, the Project Team reviewed the scientific and grey literature to identify the main documents reporting on patient‐centredness in health care from the point of view of care providers, research organizations, citizens and patient organizations at national and international level. Relevant regional and national Italian regulations were also considered for the scope. The documents were discussed with members of the Advisory Board, who provided guidance on their use in the context of the civic evaluation of quality in hospital care. We found substantial heterogeneity in the terminology and interpretation of the concept of ‘centredness’, reflecting the background of different professional disciplines, perspectives and clinical settings, in the context of specific regional settings.^26^

Consequently, we adopted a holistic vision^10^ to define person‐centredness as ‘the commitment to orient the setting of care, diagnostic and therapeutic programmes as much as possible towards the person, considered in all inherent physical, social and psychological aspects’.^27^

### 2.2 Construction of the survey tool

The structure of the survey tool was defined between October 2011 and June 2012 as a ‘checklist’ aimed at measuring compliance to the stated principles using multiple items, whose values could be conveniently added up to compute summary scores for specific aspects of interest.

The checklist included four major areas of patient centeredness in acute care: person‐oriented organizational and care processes, physical accessibility and comfort, access to information and transparency, citizen‐patient professional relationship.

Consensus over the final structure was reached after two rounds of comments received from members of the Regional Network in terms of conceptual coherence, coverage of relevant aspects and overall clarity of the terms utilized.

A pilot test was carried out at a single hospital of central Italy in January 2012, followed by extensive application in 54 hospitals from 16 Italian Regions between February and April 2012. Several changes suggested by citizens and health professionals were later incorporated in the final version released in June 2012. The construction of the survey tool was based on participatory principles rather than the application of statistical methods on empiric data, for example factor analysis. We preferred embracing a process of knowledge sharing between citizens and health professionals to incorporate stakeholder involvement^28^ and expert opinion in the measurement of person‐centredness, in order to enhance the sense of ‘community ownership’ of the evaluation procedure.^29^

The final ‘checklist’ adopted for the national survey included the above mentioned 4 areas, subdivided into 12 sub‐areas, 29 criteria and 243 items. The majority of items were dichotomous, indicating presence/absence of selected characteristics (coded as 0/10), except for cases where the questions allowed ordinal responses (coded from 0 to the number of levels). Missing data were not allowed, except for items classified as ‘not applicable’.

An example of items, scores and explanatory notes included in the checklist is shown in Table 1. Briefly, the value assigned to each item was based on the examination of different type of materials: documents (‘DOC’), direct observation (‘OBS’) or both (‘DOC/OBS’), along with additional explanatory notes.

**TABLE 1:** Examples of items used for the compilation of the checklist

Criterion	Item	Data collection method	Coding (answer)	Explanatory notes
1.1.1 Psychological support facilities	4. Psychological support for children/adolescents admitted in ordinary hospitalization	DOC	10 = Yes, psychological support includes an intervention aimed at both children/adolescent and their relatives, during the entire stay in hospital 7.5 = Yes, psychological support is aimed at both children and their relatives, but only on request 5 = Yes, psychological support is provided but only for children on request 0 = No NA = Not Applicable: there are not Pediatric Units for ordinary hospitalization inside the facility	Only for facilities providing hospitalization in Pediatric Units, in ordinary hospitalization or day hospital (DH), to users from 30 d old to 14/18 y (depending on the different local contexts). Service can be provided by the facility or in partnership with associations, cooperatives, etc within specific written agreements ‘The entire stay in hospital’ includes at least three phases: the reception phase, in which the role of the psychologist is to accompany the patient during the phase of clinical investigation and communication of diagnosis; phase of support before surgery, where the primary objective is to contain the anxiety and fear associated with the separation between parents and child; ‘rehabilitation’ phase and support in the moment of greatest post‐operative debilitation.
1.2.2 Respecting confidentiality	39. Rooms reserved for terminally ill patients and for their assistance by relatives are ensured in the General Medicine Unit compatibly with the structural resources	DOC	10 = Yes 0 = No NA = Not Applicable, there is not a General Medicine Unit inside the facility	Take the General medicine Unit as a reference. If there are more Units of General Medicine, take as a reference the Unit with the highest number of beds. The assurance of a room for terminally ill patients must be written in one of the documents delivered to patients (Service Charter, leaflet of the Unit) or foreseen within an operative procedure
	41. Rooms with ‐ partial or total ‐ visual separation between beds in the General Surgery Unit	OBS	<n> = Number of rooms with ‐ partial or total ‐ visual separation between beds in the General Surgery Unit /Overall number of rooms in the General Surgery Unit NA = Not Applicable: there isn't General Surgery Unit in the facility [0‐10] ordinal scale (10 = complete fulfillment of requirement; 0 = absence of requirement)	Visual separation means any device ‐ mobile or fixed ‐ that prevents the partial or total view of the patient hospitalized in the next bed, promoting confidentiality (for example, a curtain, a separé, etc) Do not consider any single rooms in the calculation If there are more Units of General Surgery, take the Unit with the highest number of beds as a reference
3.2.3 Website contents and accessibility	207. On the hospital website there are alternatives equivalent to audio and/or visual content	DOC	10 = Yes 0 = No	To guarantee the website accessibility to people with disabilities (blind or partially sighted, with deafness and hearing loss, learning difficulties, cognitive limitations, limited freedom of movement), in compliance with the law n. 4 of 9 January 2004, on ‘Measures to promote the access of disabled people to IT tools’ (Stanca law).

The details of all components of the checklist, including the description of the single items and the range of their possible values, are attached to the present publication as Appendix S1.

We further assessed the level of correlation between criteria, sub‐areas and areas included in the checklist to ensure that no redundant items were included (results not shown).

### 2.3 Survey design

**FIGURE 1:**
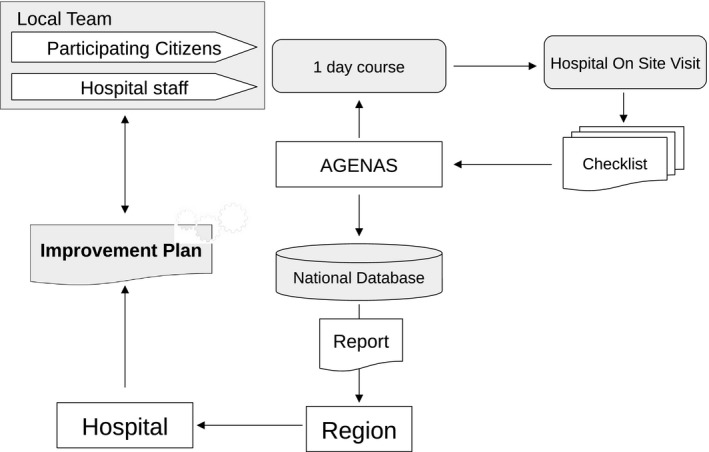
Participatory procedure for data collection

The protocol of the data collection procedure included a series of steps, underpinned by three guiding principles: empowerment of citizens and health professionals, humanization of care and continuous quality improvement (see Figure 1).

Each participating Region/Autonomous Province was requested to form a Regional Coordination Group, including members of the Regional Network, supported by regional referents of hospital managers, professionals and citizens. The Regional Coordination Group was put in charge of enrolling hospitals and coordinating the survey in each region, while hospitals were requested to form a Local Team, including local referents of health professionals and citizens.

The Project Team provided each Regional Coordination Group with standardized training materials including the programme, slides and guides for implementing the protocol. Subsequently, the Regional Coordination Group organized 1‐day training sessions in each region to induct Local Teams on the rationale of the study and the content of the checklist, while showing how to fill sheets during hospital visits on site. Following the course, each Local Team organized a series of activities, to help citizens and health professionals familiarizing with the checklist and the documents required to fill the items. Subsequently, Local Teams organized 1‐day visits at hospitals, according to their preferred schedule. The procedure relied on the observation of normal activity run in hospital wards. Hospital staff were informed of the conduct of the survey and asked to provide any additional documentation required to complete the checklist.

At the end of each visit, a final briefing was held on the same day or immediately afterwards. During this briefing, local referents of professionals and citizens discussed the contents of the checklist, assigning a unique score to each item. Once completed and quality checked, the checklist was transferred to a web platform maintained by AGENAS, where all records were finally processed. Descriptive reports were sent back to the Regions/Autonomous Provinces, where the Local Teams used the results to co‐design data‐driven hospital improvement plans.

### 2.4 Data collection and statistical analysis

In September 2016, AGENAS launched a call inviting all Regions/Autonomous Provinces to conduct a survey of patient‐centredness of accredited public and private hospitals. Accepting Regions/autonomous Provinces were invited to follow the structured process described above.

The national survey was completed between February 2017 and July 2018.

The data collection was based on standardized empty Excel sheets, manually filled in hardcopy by Local Teams at the end of their visit at hospitals and then transferred in electronic format to the central database maintained by AGENAS, where all statistical analyses were carried out. The central database included all filled items of the checklist, merged with general characteristics of hospital case mix including the Regions/Autonomous Provinces, province, type of hospital and number of beds, available from official statistics.^30^

All summary scores by criteria, area and sub‐area were normalized on a numeric scale ranging between 0 and 10, with 0 corresponding to the lack or absence of requirement and 10 to complete fulfilment.

All analyses were stratified by macroregion (north, centre or south), type of hospital (public, trust, private and academic/research) and volume of activities (determined as a proxy by their quartile in the ordered ranking of number of beds).

Descriptive statistics included the calculation of percentages and medians and ranges for variables that were not normally distributed according to the Shapiro‐Wilk test.^31^

Results were stratified by area, sub‐area and overall, according to the categories above. Histograms were used to examine the distribution of scores within and between classes.

Multivariate linear regression was used to formally test the statistical significance of a unit increase in the quartile of number of beds and scores achieved by the checklist, for each area and overall, adjusted by the other main hospital characteristics previously identified.

Regression diagnostics were used to check for compliance with the assumptions of linear regression, using the Shapiro‐Wilk test for the normality of residuals, the Breusch‐Pagan test for heteroskedasticity and the autocorrelation of residuals.^32^

Significance tests were followed by sensitivity analyses of the inclusion/exclusion of selected covariates, highlighting the presence of any cluster effects that could undermine the reliability of parameter estimates. A more robust linear model incorporating intra‐cluster correlation was carried out using generalized estimating equations (GEE) with a Gaussian family, identity link and exchangeable correlation. The GEE model preserved the presentation of the results in terms of beta coefficients, which can be interpreted as the change of person‐centred scores per unit increase of independent factors, together with their 95% robust confidence intervals and p values.^33^ All analyses were carried out by AGENAS using R.^34^

## 3 RESULTS

The main characteristics of the study sample, compared to all Italian hospitals in year 2017, are presented in Table 2.

**TABLE 2:** General characteristics of the study sample

Hospital characteristic	Survey sample of hospitals		Total population of Italian hospitals^a^	
	N	%	N	%
N	387	30.1	1287	100
Region				
North	109	28.2	549	42.7
Centre	98	25.3	259	20.1
South, islands	180	46.5	479	37.2
Type of hospital				
Public	251	64.9	595	46.2
Trust	28	7.2	52	4.0
Private	59	15.2	522	40.6
Academic, research	49	12.7	118	9.2
Volume (no. beds)				
Q1 (≤105)	98	25.3	665	51.7
Q2 (>105, ≤183)	97	25.1	283	22.0
Q3 (>183, ≤339)	95	24.5	229	17.8
Q4 (>339)	97	25.1	110	8.5

^a^ Italian Ministry of Health, Hospital Statistics, 1st January 2017 (http://www.dati.salute.gov.it/dati/dettaglioDataset.jsp?menu=dati&idPag=18).

A total of 16/21 (81%) Regions/Autonomous Provinces of Italy participated to the study, accounting for 63.6% of the total population and 59.2% of the National GDP of Italy.^35^

A total of 387 on site hospital visits were conducted by 711 citizens, 294 associations and 839 professionals participating in the Local Teams. A median of 4 raters per visit was involved in the data collection, ranging between 2 and 17. The average size of the Local Team was likely to be underestimated, due to incomplete reporting of the total number of participants enrolled in multiple visits.

The survey covered nearly one third of 1,287 acute care hospitals (30.1%).

The Regions/Autonomous Provinces from the north were under‐represented (28.2% vs 42.7%), as opposed to those from the south and the islands (46.5% vs 37.2%).

Private hospitals were remarkably under‐represented (15.2% vs 40.6%), compared to hospital trusts (7.2% vs 4.0%) and public hospitals (64.9% vs 46.2%).

Compared to the survey sample, Italy presented a much higher proportion of smaller hospitals (51.7% with a maximum of 105 beds vs lower sample quartile) and considerably less in the higher end (8.5% with at least 340 beds vs upper sample quartile).

The overall score and those of individual areas included in the checklist, except for ‘transparency’, showed to be not normally distributed according to the Shapiro‐Wilk test (details reported below). Therefore, we presented all descriptive results consistently as median scores (range) for criteria, sub‐areas and overall (see Table 3).

**TABLE 3:** Checklist for the participatory assessment of patient centeredness in hospitals and median levels achieved in the survey (N = 387)

Areas	Sub‐areas	Criteria	No. items	Median (range) achieved in the survey	
1 Person‐oriented organizational and care processes	1.1 Attention to frailty and personal needs	1.1.1 Psychological support facilities	6	7.5 (0.0‐10.0)	7.8 (1.4‐10.0)
		1.1.2 Activities/actions to promote social relations and continuity to the outside world	5	5.8 (0.0‐10.0)	
		1.1.3 Facilitating relational and emotional support from family or others	11	7.5 (0.0‐10.0)	
		1.1.4 ‘Hospital without pain’	11	9.5 (0.0‐10.0)	
	1.2 Respect for privacy	1.2.1 Respecting personal anonymity and non‐disclosure of sensitive data	4	10.0 (0.0‐10.0)	6.7 (1.6‐10.0)
		1.2.2 Respecting confidentiality	5	7.0 (0.0‐10.0)	
	1.3 Commitment to guarantee cultural, ethnic and religious non‐discrimination	1.3.1 Respecting linguistic specificity	5	5.0 (0.0‐10.0)	7.4 (3.2‐10.0)
		1.3.2 Respecting religious needs	5	6.0 (2.0‐10.0)	
		1.3.3 Respecting ethnic and cultural specificity	10	5.0 (0.0‐10.0)	
	1.4 Continuity of care	1.4.1 Facilitating continuity of care	6	6.7 (1.7‐10.0)	7.1 (2.3‐ 9.8)
2 Physical accessibility, liveability and comfort of the facilities	2.1 Physical accessibility	2.1.1 Removal of architectural and sensory barriers	10	4.4 (0.0‐10.0)	6.8 (0.9‐10.0)
		2.1.2 Pedestrian and vehicles accessibility	9	8.8 (3.8‐10.0)	
	2.2 Logistics and signposting	2.2.1 Orientation and signposting	7	10.0 (0.0‐10.0)	7.8 (1.4‐10.0)
		2.2.2 Internal pathways	3	10.0 (0.0‐10.0)	
	2.3 Person‐oriented wards	2.3.1 Wards equipment and characteristics	15	6.8 (0.0‐10.0)	5.5 (1.0‐10.0)
		2.3.2 Child‐friendly wards	7	10.0 (0.0‐10.0)	
		2.3.3 ‘Hotel standard’ comfort	9	7.5 (3.3‐10.0)	
	2.4 Overall comfort	2.4.1 Comfort of common areas	8	6.2 (0.0‐10.0)	10.0 (1.1‐10.0)
		2.4.2 Comfort of waiting rooms	18	8.9 (1.7‐10.0)	
3 Access to information, streamlining and transparency	3.1 Simplified procedures	3.1.1 Simplified booking processes	19	5.8 (0.0‐10.0)	7.5 (0.0‐10.0)
		3.1.2 Simplified access to services	4	10.0 (0.0‐10.0)	
	3.2 Facilitated access to information and transparency	3.2.1 Facilitated access to clinical documentation	12	6.7 (2.7‐10.0)	7.1 (2.3‐ 9.8)
		3.2.2 Access to information	10	7.2 (0.0‐10.0)	
		3.2.3 Website contents and accessibility	8	7.5 (0.0‐10.0)	
4 Taking care of the relationship with patients and citizens	4.1 Relationship between health professionals and patients	4.1.1 Taking care of clinical communication and individual empowerment	15	6.7 (0.6‐10.0)	7.8 (1.4‐10.0)
		4.1.2 Training and supporting the staff in the relationship with patients	6	8.0 (0.0‐10.0)	
	4.2 Relationship with citizens	4.2.1 Taking on commitments towards citizens	12	8.3 (0.0‐10.0)	5.5 (1.0‐10.0)
		4.2.2 Reception	2	10.0 (0.0‐10.0)	
		4.2.3 Training the front staff	1	10.0 (0.0‐10.0)	
Overall			243	7.0 (3.2‐9.5)	

Overall, hospitals achieved moderate levels of person‐centredness, with a median score equal to 7.0 (3.2‐9.5).

In terms of criteria, the maximum median score of 10 was achieved by ‘personal anonymity’, ‘signposting and internal pathways’, ‘child‐friendly wards’, ‘simplified access to services’ and ‘reception’. A high mark was also reached for ‘taking on commitments towards citizens’.

However, scores for all the above criteria were also quite variable, ranging from 0 to 10.

Criteria where scores were at least one mark below the overall median include ‘actions to promote social relations’, ‘respecting linguistic, ethnic and cultural specificity’, ‘removal of architectural and sensory barriers’, and ‘simplified booking processes’. No criteria achieved median scores equal or exceeding one mark over the overall median.

Scores were less variable at the macrolevel of sub‐area, which could be explained by the fact that hospitals did not consistently achieve systematically higher or lower scores across different criteria. As the sums involved a higher number of items, results over multiple criteria had a tendency to regress towards the mean. The maximum median score was achieved by ‘overall comfort’.

The sub‐areas of ‘person‐oriented wards’ and ‘relationship with citizens’ presented a median at best one mark below the overall median.

The level of correlation between sub‐areas showed levels of correlation between 0.09 and 0.64, confirming the relevance of all items included in the checklist (detailed results not shown).

Table 4 shows the distribution of area scores for specific categories of hospitals and overall. Median scores achieved for single areas did not differ substantially from the overall median, although ‘physical accessibility’ achieved a clearly higher score (7.4, 2.7‐9.7), immediately followed by ‘relationship’, which was also very variable (7.3, 0.8‐10.0).

**TABLE 4:** Scores achieved (median, range) for specific categories of hospitals, by centeredness area and overall

Hospital characteristic	1. Care processes	2. Access	3. Transparency	4. Relationship	Overall
All	6.8 (2.0‐9.8)	7.4 (2.7‐9.7)	6.7 (3.4‐9.5)	7.3 (0.8‐10.0)	7.0 (3.2‐9.5)
Region					
North	7.6 (4.0‐9.8)	7.8 (4.5‐9.6)	7.0 (3.8‐8.7)	7.9 (3.2‐10.0)	7.4 (5.2‐9.2)
Centre	6.8 (2.0‐9.5)	7.8 (3.8‐9.7)	6.8 (4.7‐9.5)	7.5 (2.5‐10.0)	7.1 (4.4‐9.5)
South, islands	6.2 (2.0‐9.7)	7.1 (2.7‐9.6)	6.2 (3.4‐9.4)	6.9 (0.8‐10.0)	6.6 (3.2‐9.5)
Type of hospital					
Public	6.7 (2.0‐9.7)	7.2 (2.7‐9.7)	6.8 (4.0‐9.5)	7.1 (1.6‐10.0)	6.9 (3.2‐9.5)
Trust	6.6 (3.3‐9.2)	7.2 (4.9‐9.6)	6.1 (4.1‐8.6)	7.9 (0.8‐9.6)	6.7 (4.0‐9.3)
Private	7.1 (2.9‐9.8)	7.8 (4.7‐9.1)	6.5 (3.4‐8.4)	7.3 (2.8‐10.0)	7.2 (4.1‐9.2)
Academic, research	7.3 (4.2‐9.3)	8.1 (4.8‐9.2)	6.4 (3.8‐8.9)	8.4 (4.0‐10.0)	7.7 (5.0‐8.9)
Volume (no. beds)					
≤105 (Q1)	6.1 (2.0‐9.7)	6.6 (2.7‐9.3)	6.3 (3.4‐9.4)	6.6 (0.8‐10.0)	6.4 (3.2‐9.5)
>105, ≤183 (Q2)	6.8 (3.0‐9.5)	7.5 (3.8‐9.7)	6.7 (4.0‐9.5)	7.0 (2.5‐10.0)	6.9 (4.6‐9.5)
>183, ≤339 (Q3)	6.9 (2.0‐9.5)	7.6 (4.6‐9.6)	6.7 (4.3‐8.5)	7.5 (2.4‐10.0)	7.1 (3.9‐8.9)
>339 (Q4)	7.3 (3.5‐9.8)	7.8 (5.0‐9.6)	6.9 (4.3‐8.9)	8.1 (2.6‐10.0)	7.5 (4.7‐9.3)

For specific categories, we found that northern regions presented consistently higher scores, while hospital trusts and public hospitals were consistently lower. The only exceptions were ‘relationships for hospital trusts’ (7.9, 0.8‐9.6) and ‘transparency for public hospitals’ (6.8, 4.0‐9.5).

**FIGURE 2:**
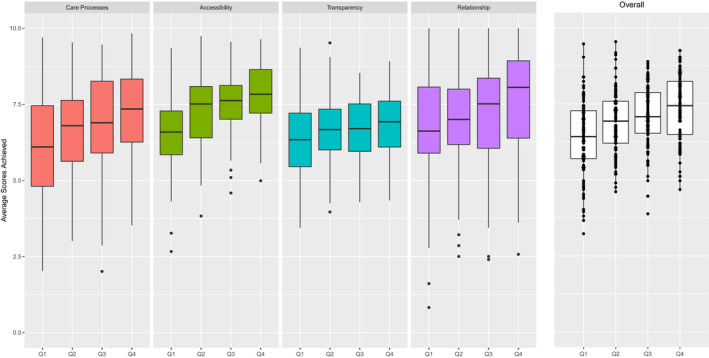
Distribution of Average Scores achieved by Italian Hospitals by Area and Overall by Quartiles of Hospital Size (No. Beds)

Hospitals in the lower quartiles of the distribution of number of beds showed invariably lower scores for all areas and overall. The examination of histograms highlighted a potentially significant linear relation (see Figure 2).

The statistical significance of this relation was formally tested in a series of multivariate linear regression models, whose compliance with fundamental assumptions showed to be problematic (see Table 5). Consistently with the non‐normality of the outcome variable, we found that the residuals were almost in all cases not normally distributed and heteroskedastic. Given the large sample, we considered non‐normality of the residuals as a minor problem and focussed more on heteroskedasticity.^36^

**TABLE 5:** Regression diagnostics for different areas of person centeredness and overall (*P Value; **Value)

Model	Test	Test *P* value/value	Access	Transparency	Relationship	Overall
Outcome variable	Shapiro‐Wilk*	<0.001	<0.001	0.30	<0.001	0.01
Multivariate linear model	Shapiro‐Wilk*	0.03	<0.001	0.19	<0.001	0.03
	Breush‐Pagan*	<0.001	0.09	<0.001	0.01	<0.001
Multivariate linear model (no outliers, no macroregion)	Shapiro‐Wilk*	<0.01	<0.01	0.83	<0.001	0.02
	Breush‐Pagan*	0.07	0.20	<0.01	0.60	0.50
	Autocorrelation**	0.45	0.27	0.31	0.37	0.42
Multivariate GEE model (Gaussian, identity link)	Alpha (exchangeable correlation)**	0.25	0.22	0.30	0.21	0.28

Sensitivity analysis showed that heteroskedasticity could be resolved by excluding the macroregions from the set of covariates. Nevertheless, there was still a moderate degree of autocorrelation between residuals, ranging between 0.27 and 0.45. Therefore, we used generalized estimating equations as an appropriate means to take into account the intra‐regional cluster effect caused by the same Local Team being involved in multiple hospital visits. The values of exchangeable correlation ranging between 0.21 and 0.30 in the GEE models confirmed this assumption. The GEE confidence intervals were considerably wider than those obtained by multivariate linear regression, turning several terms into non‐significant.

The results obtained from the application of GEE models are shown in Table 6.

**TABLE 6:** Results of GEE regression models for different areas of person centeredness and overall (N = 387)

Hospital characteristic	Care processes		Access		Transparency		Relationship		Overall	
	Estimate (95% c.i.)	*P*	Estimate (95% c.i.)	*P*	Estimate (95% c.i.)	*P*	Estimate (95% c.i.)	*P*	Estimate (95% c.i.)	*P*
Intercept	**6.76 (6.18, 7.33)**	**<.001**	**6.59 (6.28, 6.90)**	**<.001**	**6.79 (6.50, 7.08)**	**<.001**	**6.81 (6.01, 7.61)**	**<.001**	**6.69 (6.33, 7.06)**	**<.001**
Region										
North (r.c.)	0.00	–	0.00	–	0.00	–	0.00	–	0.00	–
Centre	−0.09 (−1.36, 1.18)	.89	0.39 (−0.37, 1.14)	.31	0.40 (−0.42, 1.21)	.34	0.25 (−1.12, 1.39)	.83	0.25 (−0.70, 1.19)	.61
South, islands	−**1.64 (**−**2.46,** −**0.82)**	**<.001**	−**0.78 (**−**1.21,** −**0.35)**	**<.001**	−**0.57 (**−**1.05,** −**0.09)**	**.02**	−**1.26 (**−**2.49,** −**0.04)**	**.04**	−**1.03 (**−**1.62,** −**0.45)**	**<.001**
Type of hospital										
Public (r.c.)	0.00	–	0.00	–	0.00	–	0.00	–	0.00	–
Trust	−0.04 (−0.46, 0.38)	.85	−0.05 (−0.33, 0.23)	.71	−**0.53 (**−**0.89,** −**0.17)**	**<.001**	0.15 (−1.01, 1.31)	.80	−0.13 (−0.55, 0.28)	.53
Private	0.33 (−0.32, 0.97)	.19	**0.63 (0.33, 0.93)**	**<.001**	−**0.48 (**−**0.82,** −**0.15)**	**<.01**	0.46 (−0.05, 0.96)	.08	0.22 (−0.18, 0.63)	.27
Academic, research	0.67 (−0.02, 1.36)	.06	0.43 (−0.22, 1.08)	.19	−0.32 (−0.74, 0.10)	.14	**1.04 (0.02, 2.06)**	**.04**	0.41 (−0.23, 1.04)	.21
Volume (no. beds)										
Q1‐Q4 (unit increase)	**0.21 (0.09, 0.34)**	**<.001**	**0.30 (0.24, 0.37)**	**<.001**	**0.10 (0.01, 0.20)**	**.03**	0.14 (−0.03, 0.31)	.10	**0.21 (0.13, 0.29)**	**<.001**

Results for statistically significant covariates are shown in bold.

Briefly, compared to Regions/Autonomous Provinces in the north, regions from the south presented significantly lower scores across all areas and overall (−1.03, −1.62 to −0.45). There was no significant difference found between different hospital types in terms of total scores achieved for the entire checklist. However, compared to public hospitals, hospital trusts scored significantly lower in terms of ‘transparency’ (−0.53, −0.89 to −0.17), private hospitals significantly higher for ‘access’ (+0.63, 0.33 to 0.93) but lower for ‘transparency’ (−0.48, −0.82 to −0.15), while academic/research institutions scored much higher for ‘relationship’ (+1.04, 0.02 to 2.06).

In terms of volumes of activity, after adjusting for all the above characteristics, we found that the average difference between adjacent quartiles was significantly associated with increased levels of person‐centredness across all areas, except for ‘relationship’. In particular, the linear relation was moderate for ‘transparency’ (+0.10, 0.01 to 0.20), intermediate for ‘care processes’ (+0.21, 0.09 to 0.34) and high for ‘access’ (+0.30, 0.24 to 0.37).

Overall, an increase of one quartile in terms of hospital volume was associated with an average increase in the score of patient‐centredness equal to +0.21 (0.13 to 0.29). In other terms, regardless of the region or type of hospital, the average difference between hospitals in the highest vs lowest quartile of volume of activity is equal to 60% of one mark of patient‐centredness out of a scale of ten.

## 4 DISCUSSION

The results of the first nationwide survey coordinated by AGENAS allowed responding to our initial set of research questions.

Regarding measurement of the person‐centredness of hospital care, our results suggest the applicability of a novel tool that is appropriate to use in similar settings, for the same purpose.

The checklist was applied successfully by over 1,500 collaborators who assessed hospitals in 16 distinct Regions and Autonomous Provinces, where services are differently organized and the type of facilities are rather heterogeneous, including both private and public hospitals with different size and volume of activity.

The different areas and sub‐areas addressed by the checklist have been variously explored by other studies. Aspects of continuity of care, empathy, communication, empowerment, respect and privacy have been investigated separately through different tools and methodologies.^10^, ^37^, ^38^, ^39^, ^40^, ^41^, ^42^, ^43^, ^44^ However, the complete multidimensional structure of the survey based on the holistic definition that we used cannot be easily compared to previous studies. As explained above, the particular value of the checklist is in its use as a tool to facilitate collaboration among all stakeholders.^10^, ^45^, ^46^

The scope of the survey covered multiple aspects that were measured through an objective process of observation and the examination of documents, as opposed to the personal experience of the patient during service provision.

The project demonstrated that is possible to measure person‐centredness with the direct participation of citizens, health professionals and decision makers. Such a method is not merely observational, as it involves writing co‐designed improvement plans after the delivery of reports by citizens and health professionals. Therefore, the derived measures of person‐centredness can be also actionable.^21^

However, we did not include the details of service improvement plans in the scope of the project. AGENAS is currently engaged in research aimed at defining methods and tools to monitor progress of co‐designed improvement plans. We are confident that this step will complete the creation of a National Monitoring System for Person‐Centred Hospital Care that will incorporate the participatory process as a key component of the continuous improvement cycle.

Regarding our second question on the differences observed across the country, despite an overall positive mark, we found that all scores were highly variable. Single criteria clearly showed that Italian hospitals still needed catching up with the rapid societal changes. The low marks obtained for ‘social relation’, ‘respect for cultural differences’, ‘booking processes’ and ‘person‐oriented wards and relationship with citizens’ seem to be concerning and demand specific action.

Most importantly, these results vary by location and hospital characteristics. The higher scores found for regions in the north may be even underestimated, given that the northern population was considerably under‐represented in the survey sample. Academic and private hospitals showed better access compared to public hospitals, the latter lacking also in terms of transparency, regardless of hospital size. Person‐centredness generally increased with the size of hospital, particularly in terms of access, although no single hospital performed systematically better or worse for all criteria. Indeed, many hospitals scored inconsistently across the different areas considered in the framework, raising questions for hospital managers in terms of attention for all aspects of person‐centredness.

Explaining the relation between volume and person‐centredness may be challenging. To the best of our knowledge, there is no evidence regarding the potential link between hospital size and patient centredness. On the other hand, there is evidence that lower volumes of acute care are related to poorer outcomes,^47^ with few studies exploring the link between hospitals beds and different sub‐components of centredness, for example patient satisfaction and experience of care, which showed higher scores for smaller hospitals, as opposed to the larger ones.^48^, ^49^, ^50^, ^51^

The results of our survey are therefore surprising: a possible reason could be that larger hospitals serve a more complex population and are generally more organized to deal with the different needs included in the checklist. These hospitals have also more resources available to respond to the demand of citizens for better services. On the other hand, smaller hospitals may not consider features as relevant to them, as shown by the absence of marks classified as ‘not applicable’. The impact of the different cultural background of citizens and health professionals included in the Local Teams could not be ascertained. Broader pools of experts may be needed to avoid bias in the assessment procedure.

In 2020, while preparing the present report, the outbreak of COVID‐19 hit the country with unexpected impact.^52^ Many aspects of hospital management were put under discussion, with practices being dramatically reshaped, in ways that might not revert to prior conditions soon, if ever ‐ within regions and at national level.^53^

Nevertheless, the criteria measured by the checklist correspond to universal needs or rights, towards which hospitals will have to adapt, identifying new solutions. For instance, the ‘Open‐Intensive‐Care‐Unit model’, an element measured by the checklist, might need to be revised, according to the new guidelines of patient safety. In this case, the need of the patient to maintain a relational and emotional link with the family might become even more relevant. A possible solution could be offered by information and communication technologies that could be adopted faster than before by hospitals, as naturally happened in society during the outbreak.

The checklist will need to evolve accordingly, taking also the critical area of patient safety in due account. At the time of writing, we have been testing a Appendix S1 that included 30 items, considering a number of aspects, for example the prevention of health‐care associated infections, using a surgical safety checklist, incident reporting, communication to patients and their families affected by an adverse event. There is prospect to expand and include this section after COVID‐19 as a permanent feature of the checklist. Currently, this activity lies within the mandate of the National Observatory of Good Practices for Patient Safety set by a national law, coordinated by AGENAS in collaboration with the Ministry of Health and the Regions/Autonomous Provinces.^54^, ^55^ A recent call for practices on COVID‐19 has already collected significant experiences that could be used for the scope.^56^ Lessons learned from COVID‐19^57^ solicit an immediate effort to focus participation on a broad range of community services, for example primary care, rehabilitation, residential care, home care and mental health‐care services. Targeted investments will be needed to strengthen these services through a Participatory National Program that can enable person‐centredness as an integral component of the national health system.^58^, ^59^

Finally, some relevant limitations of the study are worth to be outlined.

Firstly, the instrument has not been formally validated in terms of reliability. As explained in the methods section, we based the construction of the checklist on stakeholder engagement. However, the routine use of the survey tool across the country would require adapting its contents to a broader range of evaluators, which might be more feasible with a reduced number of essential items. Specific techniques could be used for the scope, for example inter‐rater agreement and correlation analysis, to highlight controversial and/or redundant items that can be excluded from the final version.

Secondly, the selection of hospitals was based on the subjective judgement of Regional Coordination Groups, whose participation to the survey was voluntary. This could generate bias, without guaranteeing a representative sample of Italian hospitals. The study did not collect sufficient information on private hospitals, which are more present in specific regions. However, the high level of participation has provided valid indications on future developments.

Thirdly, the scoring mechanism adopted for criteria, sub‐areas and areas was not normalized according to a standardized method, but empirically based on data collection. However, the summary measures were intuitive and the observed variability was based on a large sample. Further use of this method beyond hospital care may improve the general validity of the approach.

Finally, a risk adjustment model was not specified at the outset to ensure fair comparisons. However, the multivariate model was not estimated to justify different scores achieved for specific contexts. Our approach was aimed at testing the significance of any relation, for example the independent association between hospital size and total scores of patient centredness, taking the main potential confounders into account. Further work will be required to collect all relevant characteristics, within improved information infrastructure^60^ to collect data in compliance with the most current legislation on privacy and data protection.^61^

## 5 CONCLUSION

We conducted a nationwide participatory programme for the evaluation of person‐centredness in Italian hospitals, identifying a checklist for repeated use that can be periodically updated to improve the overall quality of acute care.

Results obtained in 16 out of 21 Regions and autonomous Provinces were overall moderately positive, although critical areas were identified, showing a substantial variability of implementation within and between hospitals. Lower levels of person‐centred care were reported by hospitals in the south and the islands, as opposed to the north and those with a higher volume of activity.

The progress of incorporating person‐centredness in the Italian SSN faces the challenges of transforming health care after Covid‐19 in the complex scenario of decentralized governance.^52^, ^62^

The necessary link with patient safety and co‐designed planning may represent a useful learning process for other countries experiencing the same problems.

## ACKNOWLEDGEMENTS

The present paper would have not been produced without the proactive involvement of 177 representatives from civic associations, healthcare facilities and 21 Italian Regions and Autonomous Provinces. These precious collaborators provided help with the development of the checklist and the conduction of the national participatory assessment of person‐centred hospital care. We are particularly grateful to the following members of the Advisory Board, from Cittadinanzattiva: Tonino Aceti, Michela Liberti, Rosapaola Metastasio, Francesca Moccia, Sabrina Nardi, Alessio Terzi, Maria Vitale. We thank the following representatives of the R&AP from the Regional Network (RN) and the Regional Coordination Group (RCG): Lidia Bocci, Maria Assunta Ceccagnoli, Aldo Cerulli, Bruno Cipollone, Laura Ottaviani, Santina Scassa, and Luigia Calcalario, Angelo Muraglia (Regione Abruzzo); Angela Pia Bellettieri, Maria Antonietta Tarsia and Gabriella Sabino (Regione Basilicata); Haimo Kaser (Autonomous Province of Bolzano‐Bozen); Monica Loizzo, Rossana Maida, Caterina Tavano, Michelangelo Iannone, Giuseppe Afflitto, Gabriella Lampasi and Francesca Fratto, Giuseppina Russo (Regione Calabria); Teresa Angiello, Renato Pizzuti, Cinzia Rea (Regione Campania); Giovanni Ragazzi, Michele Dal Pozzo, Ottavio Nicastro, Fabrizio Rubino, Giovanna Campaniello, Maria Puddu, Cristiana Damini, Viola Damen, Giuseppina Bergamini, Laura Biagetti, Filippo Caniglia, Raffaella Barresi, Francesca Bravi, Giorgia Valpiani, Nunzia Boccaforno, Alessandra Affatato, Giuseppina Poletti, Gabriella Fabbri, Remo Piera Nobili, Claudia Reggiani and Vittoria Sturlese and Maria Augusta Nicoli (Regione Emilia‐Romagna); Anna Paola Agnoletto, Barbara Lavia, Francesca Tosolini (Regione Friuli‐Venezia Giulia); Alessandro Bazzoni, Maria Maddalena Sanna, Arturo Di Folco, Gianna Sangiorgi, Sergio Tomaino, Mario Braga and Valentino Mantini, Angelo Tanese, Gianni Vicario, Carmen Mantuano (Regione Lazio); Anna Verna, Lucia Vinci Barbieri, Enrica Orlandini, Alessandra Moisello and Giorgia Auteri, Rosa Bellomo (Regione Liguria); Giancarlo Fontana (Regione Lombardia); Silvia Rossi, Roberto Amici, Renza Barbon, Monia Mancini, Maria Rita Materazzi, Maria Luisa Quaglieri, Alberto Deales and Francesco Di Stanislao, Deborah Gordini, Cecilia Palazzesi (Regione Marche); Paola Sabatini, Stefania Pizzi, Mario Vitarelli, Sandra Scarlatelli, Giuseppe Cerere and Lolita Gallo, Marinella D’Innocenzo, Francesco Colavita (Regione Molise); Angelo Penna, Paola Borelli, Italia Di Marco, Rosanna Cerri, Alessandra D’Alfonso, Giuseppina Viola, Teresa Giachino Amistà, Giorgio Pretti, Elisabetta Sasso, Marco Rapellino and Mirella Angaramo (Regione Piemonte); Michele Muci, Antonella Celano, Stefania Palmisano, Ambrogio Aquilino and Sonia Giausa, Giovanni Gorgoni (Regione Puglia); Antonella Virdis, Franca Billa, Bruna Dettori, Grazia Cattina, Michele Golino, Daniela Medda, Massimo Timussi, Donatella Garau and Rita Pilloni, Maria Paola Pilloni (Regione Sardegna); Gelsomina Di Pietro, Giuseppe Greco, Pieremilio Vasta, Caterina Lo Presti, Rosaria Licata, Salvo Favitta, Simona Di Virgilio and Giuseppe Murolo (Regione Sicilia); Marco Menchini, Mariangela Castagnoli, Elio Mugnaini, Aurelio Pellirone, Claudio Sarti, Ada Macchiarini, Giuseppe Notaro and Maria Teresa Mechi, Roberta Bottai, Annalisa Berti (Regione Toscana); Adriano Passerini, Merirosa Pederzolli, Daniela Bonaldi and Emanuele Torri (Autonomous Province of Trento); Manuela Pioppo, Franco Santocchia, Luisella Pieri, Mario Amico, Rosita Morcellini, Paola Weber, Danilo Bellavita, Antonio Perelli, Linda Richieri and Gianni Giovannini, Maria Concetta Patisso, Kathryn Mahan (Regione Umbria); Marco Ottonello, Sabrina Ghidoni, Assunta Dodaro, Roberto Novati and Patrizia Vittori (Regione Valle d’Aosta); Paola De Polli, Maria Cavazzin, Federico Boi, Pamela D’Incà, Simone Cristina Potì, Lorenzo Bulegato, Paolo De Pieri, Mario Saia, Manuela Miorin, Sonia Carollo, Imelda Romano, Eleonora Capovilla, Fabiola Fabris, Alessandro Lomeo, Giuseppe Cicciù, Lorenzo Signori, Antonio Maritati and Costantino Gallo, Antonella Giorgia Becchetti (Regione Veneto). Special thanks go to Beatrice Cerilli for the fundamental organizational support to the Project Team.Bradaiolo,Martelli

[Correction added on 8 July 2021, after first online publication: Acknowledgement section has been added.]

## CONFLICT OF INTEREST

The authors declare that they have no conflict of interest in relation to the production of the submitted paper.

## AUTHOR CONTRIBUTIONS

All authors provided a substantial contribution to the development and implementation of the method as well as the production of the manuscript. FlC, SC, GD, BL, AL, MC and GC designed the survey questionnaire and led the study on behalf of AGENAS. GD and FC conducted the statistical analysis for the paper. FC led the production of the paper, drafting its initial version. All authors revised and completed the production of the manuscript, revising and agreeing on its present contents.

## PATIENT AND PUBLIC INVOLVEMENT

This study has been conducted with the direct participation of policymakers, patients and the public, as described in the section on materials and methods and the acknowledgement section.

## DISSEMINATION

The results of this study have been included in extended format in a series of interim and final national report, coordinated by AGENAS, which has been circulated among all participants, including relevant patient and public communities, as fully documented in the acknowledgement section.

## Supporting information

App S1

## Data Availability

The data that support the findings of this study are available from AGENAS upon reasonable request.
